# Differences Between Patients With Chronic Epipharyngitis With and Without Previous COVID-19 Infection

**DOI:** 10.7759/cureus.51543

**Published:** 2024-01-02

**Authors:** Manabu Mogitate

**Affiliations:** 1 Otolaryngology, Mogitate ENT Clinic, Kawasaki, JPN

**Keywords:** outcome, naso-pharynx, infectious disease, endoscopy, coronavirus

## Abstract

Objective

Epipharyngeal abrasive therapy (EAT) is effective in patients with chronic epipharyngitis who previously had coronavirus disease 2019 (COVID-19). The study aimed to evaluate differences between patients with chronic epipharyngitis with (long COVID) or without a history of COVID-19 (non-long COVID).

Methods

This is a retrospective study based on the analysis of medical records of patients who visited the Mogitate ENT Clinic in Kawasaki, Japan, for six months from March 2022.

Results

Patients with long COVID were significantly younger (p=0.018). Fatigue and brain fog were prevalent in patients with long COVID, whereas throat discomfort, postnasal drip, and sputum were more common in those with non-long COVID. Epipharyngeal endoscopic findings in patients with long COVID showed significantly higher scores (p<0.001) compared with patients with non-long COVID.

Conclusions

The primary differences between patients with long COVID and non-long COVID were age, symptoms, and severity scores of endoscopic findings. The EAT should be aggressively implemented in patients with chronic epipharyngitis with or without COVID-19 infection, as there is no reason not to treat a patient with a condition caused by COVID-19 infection.

## Introduction

Patients with post-severe acute respiratory syndrome coronavirus 2 (SARS-CoV-2) infection can show some symptoms, such as general fatigue, even after the infectivity disappears. These comprise what is called the post-COVID-19 condition (long COVID); these symptoms can persist for more than two months after infection [1]. Unlike patients with SARS-CoV-2 infection who develop pneumonia and require hospitalization, most patients with long COVID who do not require hospitalization show similar symptoms to patients with chronic epipharyngitis [2].

Recently, increasing reports have indicated that long COVID complications, like chronic epipharyngitis, for which treatment with epipharyngeal abrasive therapy (EAT) appears effective [2,3]. In addition, myalgic encephalomyelitis/chronic fatigue syndrome (ME/CFS) is an often-mentioned symptom of long COVID [4]. Imai et al. [2] specifically examined whether EAT effectively treats symptoms similar to ME/CFS, such as fatigue, headache, and attention disorder. In addition, Ito reported that EAT improved subjective symptom scores in 31 patients with long COVID [3]. Currently, a prospective study on EAT use for long COVID is being conducted by the Epipharyngeal Abrasion Therapy Review Committee of the Japan Society of Stomato-pharyngology [5].

It is unclear whether there are differences between patients with chronic epipharyngitis with or without long COVID (referred to as non-long COVID). Thus, to address this question, the Mogitate ENT Clinic in Kawasaki, Japan, opened a specialized outpatient center for chronic epipharyngitis in 2016 [6]; the author retrospectively evaluated whether chronic epipharyngitis with long COVID has different characteristics to conventional chronic epipharyngitis.

## Materials and methods

Participants and study setting

The study was conducted under the tenets of the Declaration of Helsinki and authorized by the Ethics Review Committee of Ota General Hospital (approval number 22027). As there is no ethics committee at the Mogitate ENT Clinic, the author submitted an experimental protocol for this study to the ethics committee of Ota General Hospital and received confirmation that there are no ethical issues. A total of 329 patients who visited the clinic for six months starting from March 2022 were enrolled in the study. Patients diagnosed with chronic epipharyngitis or treated for EAT at other hospitals and clinics were excluded. All patients provided written informed consent. This retrospective study was based on patient medical records.

Epipharyngeal abrasive therapy (EAT)

At the initial examination, other organic diseases of the pharynx and larynx were discarded by endoscopy (endoscopy system 2022, HOYA Corporation, Tokyo, Japan; video processor EPK-i7010, and video nasal scope, VNL11-J10). The diagnosis of chronic epipharyngitis was made using white light. Briefly, after anesthetizing the nasal cavity with 1% xylocaine, an endoscope was inserted via the right nostril. The epipharyngeal endoscopic findings obtained with white light were scored according to the EAT Review Committee's assessment criteria [5] for the coloration and swelling of the epipharyngeal mucosa and for mucus or crust adhesion, which were rated on a three-point scale (0 - none, 1 - mild-moderate, 2 - severe). Then, a swab containing zinc chloride (ZnCl2) was inserted through the left nostril before inserting the endoscope via the right nostril. EAT of the entire epipharynx was conducted in patients bleeding during EAT, providing a diagnosis for chronic epipharyngitis. The degree of bleeding was similarly assessed using a three-point scale.

Patients who had some symptoms after COVID-19 infection were defined as patients with long COVID, whereas patients with usual chronic epipharyngitis who had no history of COVID-19 infection and visited for treatment were defined as patients with non-long COVID.

Study items included the following 11 items: the proportion of patients with chronic epipharyngitis and long COVID who visited the clinic for close examination and treatment; monthly visits of patients with long COVID and patients with chronic epipharyngitis but without long COVID; time of SARS-CoV-2 infection in long COVID patients; time (number of days) of long COVID before visiting the clinic; gender differences between long and non-long COVID patients; age distribution of long and non-long COVID patients; differences in chief complaints between long and non-long COVID patients. The chief complaints were divided into three pathological conditions, namely, symptoms via autonomic disturbance, direct symptoms due to epipharyngitis, and immune-mediated secondary disease as focal inflammation of chronic epipharyngitis, as reported by Hotta et al. [7]. Items included differences in the severity scores of endoscopic findings between long and non-long COVID patients; the presence or absence of pain awareness at the time of the first endoscopic EAT, and pain duration in long and non-long COVID patients; outcomes in long and non-long COVID patients, particularly, the number of drop-out cases.

Statistical analysis

Analyses were performed using SPSS version 22.0 (IBM, Inc., Armonk, US); p<0.05 indicates a statistically significant difference. Fisher's exact test was used to evaluate the number of patients in the long and non-long COVID groups per month, the number of patients in each category per month, gender differences between long and non-long COVID, difference between the main complaints of long and non-long COVID, difference in chief complaints divided into the three pathologies of chronic epipharyngitis as reported by Hotta et al. [7], and pain during the first endoscopic EAT and pain duration, number of drop-out cases, and initial drop-out cases. A Poisson distribution was used to assess the time of SARS-CoV-2 infection in long COVID patients. A one-way chi-square goodness of fit test was used to assess the number of days between the onset of SARS-CoV-2 infection and the visit to the clinic. The Mann-Whitney U test was used to analyze the age of patients with long and non-long COVID, differences in severity scores of endoscopic findings between long and non-long COVID, and the median duration of pain during the first endoscopic EAT between long and non-long COVID. The log-rank test was used to analyze the outcome of the number of drop-out cases.

## Results

A total of 329 patients were included in this study (121 males and 208 females) with a median age of 42 years (ranging from 34 to 54 years). Out of the total, 107 patients had long COVID (median age of 43 years with range: 34-56), and 222 patients had non-long COVID (median age of 40 years with range: 33-47). The incidence of chronic epipharyngitis in patients with long COVID was 100%. Significantly more patients had long COVID in July and August of 2022 compared to March (April) and June of 2022 (p=0.001). Based on the number of patients in December 2021, there were significantly more cases of SARS-CoV-2 infection in July 2022 (p=0.039). Assuming an even distribution of patients, significantly more patients visited the clinic in the first two weeks and one month after COVID-19 onset (p<0.001).

**Figure 1:**
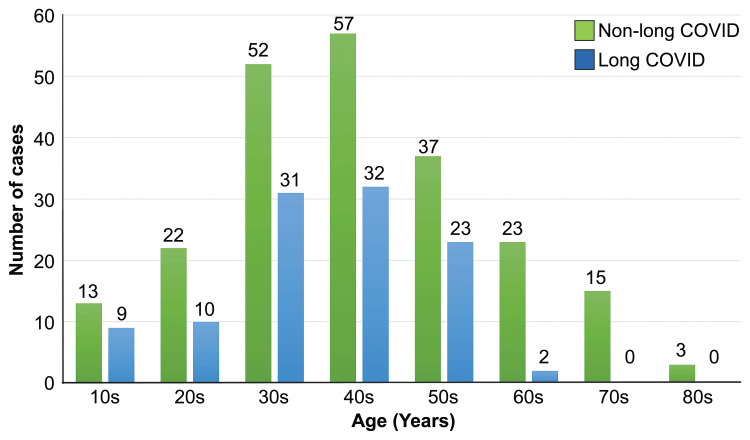
Differences in age distribution between long COVID and non–long COVID patients

There were no significant differences between gender groups. Patients with long COVID were significantly younger (p=0.018). This is because no patients had long COVID in their 70s and 80s. So, there was no significant difference except for those in their 70s and 80s (Figure 1).

**Figure 2:**
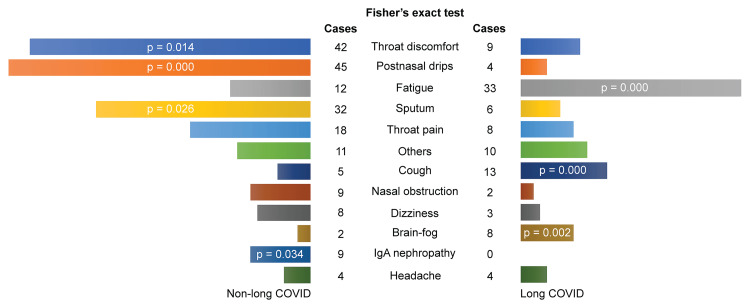
Differences in chief complaints of long and non–long COVID A p-value is mentioned only when there is a statistically significant difference (p<0.05).

Fatigue, cough, and brain fog were significantly more frequent symptoms in long COVID patients, while throat discomfort, postnasal drip, sputum, and IgA nephropathy were significantly more common in non-long COVID patients (Figure 2). Symptoms via autonomic disturbance were significantly more common in long COVID patients (p<0.01), whereas direct symptoms due to epipharyngitis (p<0.01) and immune-mediated secondary disease as focal inflammation (p=0.018) were more common in non-long COVID.

**Figure 3:**
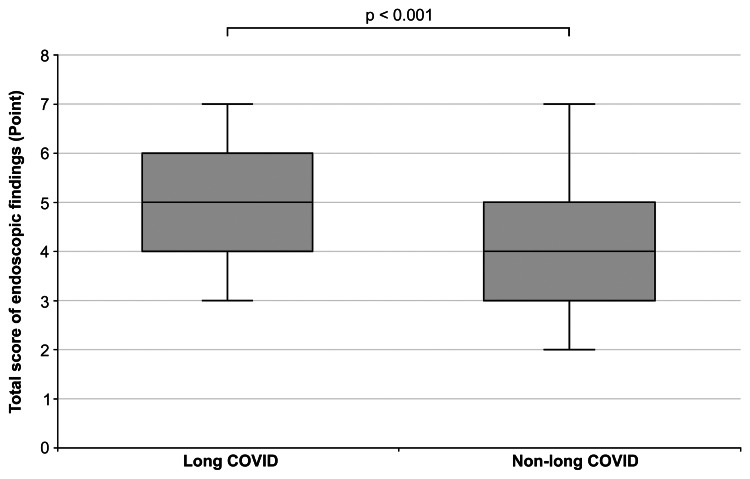
Comparison of the total score of endoscopic findings for long and non–long COVID patients

Hotta et al. [7] reported that chronic epipharyngeal symptoms have three main pathologies: (1) inflammation-mediated local symptoms, (2) neuroendocrine symptoms, and (3) autoimmune-related symptoms. Patients with long COVID were significantly more likely to have neuroendocrine symptoms, whereas those with non-long COVID were significantly more likely to have inflammation-mediated local and autoimmune-related symptoms. Figure 3 shows a comparison between the total score of endoscopic findings for the long and non-long COVID patients. The epipharyngeal endoscopic findings in long COVID (5, range: 4-6) had significantly higher scores than in non-long COVID (4, range: 2-5) with a p-value of <0.001.

A total of 160 cases, 57 Long COVID and 103 non-long COVID, were studied. Although the author asked all patients about their pain, not all had information about their pain in their medical records: only 53.3% (57 of 107 patients) with long COVID, and 46.4% (103 of 222) with non-long COVID had indication about the pain. Pain awareness and pain duration in the initial endoscopic EAT are shown in Table 1. No significant difference was found between the long and non-long COVID groups.

**Table 1:** Pain duration in the initial endoscopic epipharyngeal abrasive therapy IQR - interquartile range

Pain awareness	Long COVID n=57	Non–long COVID n=103	p-value
Yes vs. no	61 vs. 32	117 vs. 82	0.125
Median (IQR)	5 (3, 24)	8 (3, 24)	0.364

**Figure 4:**
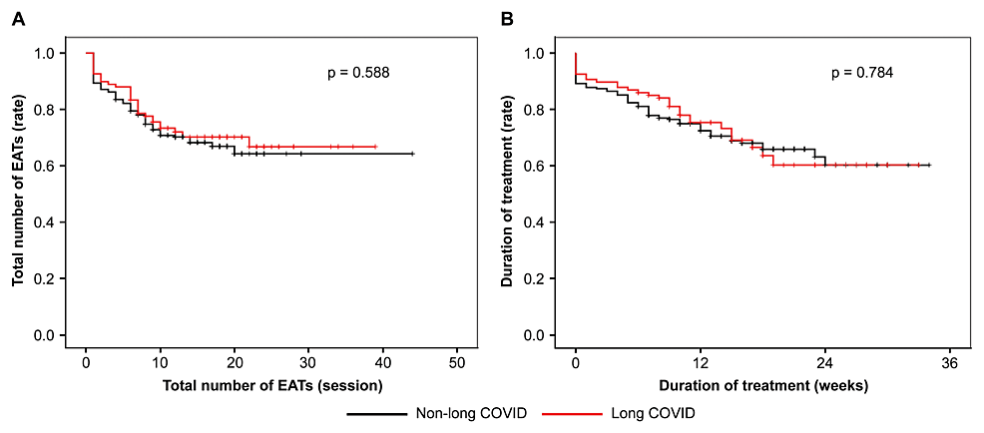
Outcomes and drop-out A: shows the drop-out rate or treatment withdrawal rate based on the number of treatments. B: shows the drop-out rate based on the duration of treatment. The follow-up period comprised the total number of EATs and treatment period (p=0.588 and p=0.784, respectively) with no significant differences between the groups. EAT - epipharyngeal abrasive therapy

Outcomes, particularly drop-outs, are shown in Figure 4. There was no significant difference between the two long and non-long COVID groups. In other words, the two groups had no difference in pain complaints.

## Discussion

In Japan, the sixth wave of SARS-CoV-2 infection, primarily due to coronavirus mutant strain BA.1, started in January 2022, while the seventh wave due to coronavirus mutant strain BA.5 took place in July 2022 [8]. Long COVID was considered to be for more than two months after SARS-CoV-2 infection [1]; therefore, this study started in March 2022 after the beginning of the sixth wave.

Regarding the incidence of chronic epipharyngitis in long COVID, Imai et al. [2] and Ito [3] reported that 93.5-100% of patients had chronic epipharyngitis. In the present study, the incidence was 100%. A major target of SARS-CoV-2 is the epithelial mucosa of the epipharynx [9,10], and it is thought that a chronic inflammatory condition exists in the epipharynx after the acute phase; it is chronic epipharyngitis, and spike proteins remain in the epipharynx several months after onset, causing a recurring inflammation [11].

The number of long COVID patients visiting the clinic was considerably higher in July and August than between March and June 2022. In addition, compared to December 2021, there were significantly more cases of SARS-CoV-2 infections in July 2022. This is most likely to be linked to the number of new cases of COVID-19. Just this time of year is known as the seventh wave in Japan. A questionnaire-based follow-up study of 1066 patients diagnosed with COVID-19 and hospitalized in Japan reported ≥1 sequelae symptoms in approximately 30% of all affected patients at 12 months [11]. The greater the number of newly affected patients, the greater the number of patients with long COVID.

Fatigue, cough, and brain fog were significantly more common in long COVID, whereas throat discomfort, postnasal drip, sputum, and IgA nephropathy were significantly more common in non-long COVID. Patients with long COVID were significantly more likely to have neuroendocrine symptoms, while non-long COVID patients were significantly more likely to have inflammation-mediated local and autoimmune-related symptoms. Ito [3] reported that the improvement in long COVID cases using EAT may be due to the improvement of cerebral microcirculation with the scraping and phlebotomy of the pathological mucosa of the epipharynx using EAT and the improvement of brain function with the autonomic nerve stimulation effect [12]. However, he also stated that long-term treatment may be necessary to improve autonomic neuropathy and brain dysfunction. In our previous study using exhaled nitric oxide (NO) testing [13], EAT, regardless of symptoms, lowered exhaled NO levels, with a concomitant reduction in patient symptoms. The difference in symptoms between the two groups is not a reason not to perform EAT.

Imai et al. [2] reported that >90% of long COVID patients show moderate to severe epipharyngeal endoscopic findings. This study compared the severity of endoscopic findings in long and non-long COVID patients and showed that significantly more long COVID (80%) than non-long COVID (64%) patients had moderate to severe epipharyngeal endoscopic findings. In addition, compared to non-long COVID, the epipharynx of long COVID patients had significantly worse inflammatory findings; thus, SARS-CoV-2 may result in increased epipharyngeal inflammation [14]. However, EAT is very effective even when the endoscopic findings are severe; we should aggressively perform EAT regardless of the endoscopic findings.

The diagnosis of epipharyngitis is based on abrasion cytology and is defined as one where the patient is aware of pain [15]. Pain is one of the reasons why patients discontinue treatment, with an approximate 5% reported drop-out rate [3]. In particular, in a prior outpatient specializing clinic in chronic epipharyngitis, approximately 20% of patients dropped out [6]. This treatment cannot be completed in one session but requires 10-20 ongoing treatments. To increase the patient's cure rate, the treatment must be free of pain and other distresses. In this study, there were no significant differences in pain perception or duration between patients with long and non-long COVID. Regardless of the cause of chronic epipharyngitis, the author believes that the cause of onset is irrelevant to the perception of pain.

This research has several limitations, namely, the small number of cases and the single-center study design. Furthermore, the patient follow-up period was short. Thus, in the future, larger studies should assess patient outcomes in a multicenter setting, including a longer follow-up period.

## Conclusions

The main differences between patients with long COVID and those with non-long COVID were age, symptoms, and severity scores of endoscopic findings. EAT should be aggressively implemented in patients with chronic epipharyngitis with or without COVID-19 infection, as there is no reason not to treat a patient with a condition caused by COVID-19 infection.
